# Extensive Anti-CoA Immunostaining in Alzheimer’s Disease and Covalent Modification of Tau by a Key Cellular Metabolite Coenzyme A

**DOI:** 10.3389/fncel.2021.739425

**Published:** 2021-10-15

**Authors:** Tammaryn Lashley, Maria-Armineh Tossounian, Neve Costello Heaven, Samantha Wallworth, Sew Peak-Chew, Aaron Bradshaw, J. Mark Cooper, Rohan de Silva, Surjit Kaila Srai, Oksana Malanchuk, Valeriy Filonenko, Margreet B. Koopman, Stefan G. D. Rüdiger, Mark Skehel, Ivan Gout

**Affiliations:** ^1^Queen Square Brain Bank, UCL Queen Square Institute of Neurology, London, United Kingdom; ^2^Department of Neurodegenerative Disease, UCL Queen Square Institute of Neurology, London, United Kingdom; ^3^Department of Structural and Molecular Biology, University College London, London, United Kingdom; ^4^MRC Laboratory of Molecular Biology, Cambridge Biomedical Campus, Cambridge, United Kingdom; ^5^Department of Molecular Neuroscience, Faculty of Brain Sciences, Royal Free Campus, London, United Kingdom; ^6^Reta Lila Weston Institute of Neurological Studies, University College London, London, United Kingdom; ^7^Department of Cell Signaling, Institute of Molecular Biology and Genetics, Kyiv, Ukraine; ^8^Cellular Protein Chemistry, Bijvoet Center for Biomolecular Research, Utrecht University, Utrecht, Netherlands; ^9^Science for Life, Utrecht University, Utrecht, Netherlands

**Keywords:** Coenzyme A, protein CoAlation, neurodegeneration, Alzheimer’s disease, oxidative stress, tau

## Abstract

Alzheimer’s disease (AD) is a neurodegenerative disorder, accounting for at least two-thirds of dementia cases. A combination of genetic, epigenetic and environmental triggers is widely accepted to be responsible for the onset and development of AD. Accumulating evidence shows that oxidative stress and dysregulation of energy metabolism play an important role in AD pathogenesis, leading to neuronal dysfunction and death. Redox-induced protein modifications have been reported in the brain of AD patients, indicating excessive oxidative damage. Coenzyme A (CoA) is essential for diverse metabolic pathways, regulation of gene expression and biosynthesis of neurotransmitters. Dysregulation of CoA biosynthesis in animal models and inborn mutations in human genes involved in the CoA biosynthetic pathway have been associated with neurodegeneration. Recent studies have uncovered the antioxidant function of CoA, involving covalent protein modification by this cofactor (CoAlation) in cellular response to oxidative or metabolic stress. Protein CoAlation has been shown to both modulate the activity of modified proteins and protect cysteine residues from irreversible overoxidation. In this study, immunohistochemistry analysis with highly specific anti-CoA monoclonal antibody was used to reveal protein CoAlation across numerous neurodegenerative diseases, which appeared particularly frequent in AD. Furthermore, protein CoAlation consistently co-localized with tau-positive neurofibrillary tangles, underpinning one of the key pathological hallmarks of AD. Double immunihistochemical staining with tau and CoA antibodies in AD brain tissue revealed co-localization of the two immunoreactive signals. Further, recombinant 2N3R and 2N4R tau isoforms were found to be CoAlated *in vitro* and the site of CoAlation mapped by mass spectrometry to conserved cysteine 322, located in the microtubule binding region. We also report the reversible H_2_O_2_-induced dimerization of recombinant 2N3R, which is inhibited by CoAlation. Moreover, CoAlation of transiently expressed 2N4R tau was observed in diamide-treated HEK293/Pank1β cells. Taken together, this study demonstrates for the first time extensive anti-CoA immunoreactivity in AD brain samples, which occurs in structures resembling neurofibrillary tangles and neuropil threads. Covalent modification of recombinant tau at cysteine 322 suggests that CoAlation may play an important role in protecting redox-sensitive tau cysteine from irreversible overoxidation and may modulate its acetyltransferase activity and functional interactions.

## Introduction

Presenting as the most common form of dementia, Alzheimer’s disease (AD) is a progressive disease affecting millions of people worldwide (Alzheimer, 1906; Selkoe et al., 1999; Przedborski et al., 2003). AD is characterized by cognitive deterioration, changes in behavior and psychiatric disturbances (Burns and Iliffe, 2009). The multifactorial nature of AD with various genetic, biochemical and molecular abnormalities provides a challenge for disease prevention, early onset diagnostics and the development of effective therapies. The majority of AD cases (∼95%) occur sporadically, with no obvious related risk factors (Castellano et al., 2012), whereas a small percentage of hereditary/familial AD (∼5%) are caused by mutations in genes encoding amyloid precursor protein (*APP*), presenilin-1 (*PS1*) and presenilin-2 (*PS2*). Pathological build-up of the β-amyloid peptide (Aβ) in extracellular plaques and intracellular neurofibrillary tangles (NFT) composed of hyperphosphorylated tau are hallmarks of both familial and sporadic AD (Lee et al., 2001). Other pathological features of AD include altered synaptic transmission, impaired calcium and lipid homeostasis, inflammation, mitochondrial dysfunction, and oxidative stress (Hoover et al., 2010; Huang et al., 2016; Magi et al., 2016; Kinney et al., 2018; Chew et al., 2020; Wang et al., 2020).

Mitochondrial homeostasis and function are central to maintaining healthy neurons (Mandal and Drerup, 2019). Healthy and functional mitochondria are vital in neuronal ATP production, intracellular calcium signaling, establishing membrane potential, and efficient neurotransmission. The hippocampal and cortical brain regions are especially vulnerable to mitochondrial disruption and oxidative stress, due to their high oxygen consumption and reliance on mitochondrial energy generation, in combination with inherently low levels of antioxidants and low neuronal cell repair capacity (Cenini and Voos, 2019; Lee et al., 2020). Build-up of damaged mitochondria and autophagic vacuoles is also a prominent feature in neurons of several neurodegenerative diseases, including AD (Nixon and Yang, 2012). Various factors have been found to induce mitochondrial damage, such as abnormal protein aggregates (Aβ, tau), reduced glucose metabolism, and exposure to toxic drugs, and prolonged production of reactive oxygen species (ROS) (Hashimoto et al., 2003; Guo et al., 2013; Stoker et al., 2019). At low levels, ROS may induce subtle changes in intracellular redox signaling. Increased and sustained production of mitochondrial ROS leads to irreversible damage of major cellular biomolecules such as proteins, lipids, and DNA through pathological redox reactions. An imbalance between ROS and antioxidant species triggers oxidative stress. Consequently, increased ROS-induced protein modifications, such as protein cysteine oxidation, carbonylation, *S*-glutathionylation and nitrosylation, have been reported in post-mortem AD brain tissue and AD animal models, indicating excessive oxidative stress-related damage (Sultana et al., 2009). The activity of several key metabolic and signaling enzymes as well as antioxidant proteins has been found modulated by oxidative stress and implicated in the progression of AD.

Tau is the main microtubule-associated protein in neurons. Under physiological conditions, tau promotes the assembly of tubulin heterodimers into microtubules, and stabilizes microtubule networks, which comprise the neuronal cytoskeleton (Grundke-Iqbal et al., 1986). In the brain, the level of tau expression is two times higher in gray matter than white matter (Binder et al., 1985). Alternative splicing of the tau gene *MAPT* generates six distinct molecular isoforms in adult human brain, ranging from 352 to 441 amino acids. At the N-terminus, tau isoforms differ by the presence of one or two N-terminal inserts encoded by exon 2, or exons 2, and 3 (1N and 2N, respectively). Exon 3 is present only in accompaniment of exon 2, therefore exclusion of both sequences generates tau isoforms lacking N-terminal inserts (0N). At the C-terminus, alternative splicing of exon 10 produces isoforms featuring three or four microtubule-binding repeats (MTBR) containing one or two naturally occurring cysteine residues, thus distinguishing between 3-repeat (3R) and 4-repeat (4R) tau, respectively (Goedert et al., 1989; Liu and Gong, 2008). The MTBRs are highly positively charged which facilitates their binding to negatively charged tubulin in microtubules. All six tau isoforms have been found in neurofibrillary tangles of AD (Goedert et al., 1992).

Tau is regarded as an intrinsically disordered protein (IDP) and forms random-coil conformations with some transient secondary structures, including α-helices, β-strands, and polyproline II helices (Battisti et al., 2012). It exists in monomeric, dimeric, oligomeric, and fibrillar forms which are implicated in physiological functions and in pathology (Meraz-Rios et al., 2010). Tau monomers are thought to form dimers in antiparallel fashion, involving hydrophobic interactions and/or covalent cysteine-mediated disulfide bonds. Tau dimers were found to assemble into oligomeric structures, which have the propensity to form paired helical filaments (PHFs) or straight filaments (SFs). Both PHFs and SFs can then assemble further into neurotoxic NFTs.

Recent analysis of neurofibrillary tangles from AD brain by cryo-electron microscopy revealed the ordered pairs of protofilaments comprising residues 306–378 and disordered amino- and carboxy-termini which form the fuzzy coat by projecting away from the core (Fitzpatrick et al., 2017). Generated atomic models of PHFs and SFs reveal inter-protofilament packing and how 3R and 4R tau isoforms can be assembled into the growing filament. Hydrophobic and polar interactions facilitate the anti-parallel β-sheet packing where hexapeptide ^306^VQIVYK^311^ is essential for the assembly of tau filaments. Residues ^321^KCGS^324^ of the first and ^313^VELSK^317^ of the second protofilament mediate the interaction between the two protofilaments. In contrast, the NMR structure of tau bound to microtubules reveals a hairpin conformation of residues 269–284 and 300–312 and the disordered structure of the N- and C-termini (Kadavath et al., 2015).

Post-translational modifications (PTMs) of tau have been extensively studied and found to modulate its microtubule-binding ability and aggregation. Tau is highly phosphorylated in normal brain and hyperphosphorylated in pathologies. In healthy brain, approximately 10 serine, threonine, and tyrosine phosphorylated sites on tau are commonly detected, in contrast to approximately 45 phosphorylation sites in AD brain (Wang et al., 2013; Iqbal et al., 2016). These sites of phosphorylation are predominantly located within the proline rich region and flanking the MTBRs. Abnormal hyperphosphorylation of tau in AD renders it unable to support microtubule function and promotes its dissociation from microtubules. Consequently, cytosolic tau favors the formation of tau aggregates (Lindwall and Cole, 1984; Ramkumar et al., 2018).

Tau is reversibly oxidized by ROS and reactive nitrogen species (RNS), which promote redox-mediated oxidative PTMs, including cysteine oxidation, S-glutathionylation, and nitrosylation. The MTBRs contain redox-sensitive cysteine residues, which have been implicated in contributing to microtubule binding (Martinho et al., 2018). Tau oxidation was shown to be associated with very slow MT polymerization, whereas glutathionylation of oxidized cysteines reversed this inhibitory effect (Landino et al., 2004). Tau is also known to possess intrinsic acetyltransferase activity, which requires Cys291 and Cys322 to function as intermediates in the transfer acetyl from acetyl-CoA to lysine residues during self-acetylation (Cohen et al., 2013). Moreover, lysine acetylation was shown to decrease microtubule binding and thereby promoting tau aggregation and NFT formation.

Coenzyme A is an essential cofactor in all living cells with diverse cellular functions (Lipmann and Kaplan, 1946; Leonardi et al., 2005; Davaapil et al., 2014; Srinivasan and Sibon, 2014; Theodoulou et al., 2014). The biosynthesis of CoA in prokaryotic and eukaryotic cells occurs via a conserved pathway involving enzymatic conjugation of ATP, pantothenate (vitamin B5) and cysteine (Leonardi et al., 2005). The presence of a highly reactive thiol group allows CoA to be involved in numerous biochemical reactions and to generate diverse metabolically active thioesters, such as Acetyl-CoA, Malonyl-CoA, HMG-CoA, and others (Tsuchiya et al., 2017). CoA and its thioesters are involved in critical anabolic and catabolic pathways, the regulation of gene expression via protein acetylation and the biosynthesis of neurotransmitters. In mammalian cells, CoA/CoA derivatives are predominantly sequestered in mitochondria (2–5 mM) and peroxisomes (0.5–1 mM), while cytosolic/nuclear levels are significantly lower (20–140 μM) (Leonardi et al., 2005). The intracellular levels of CoA/CoA derivatives fluctuate in cellular response to nutrients, hormones, metabolites, and stress (Theodoulou et al., 2014). Abnormal biosynthesis and homeostasis of CoA and its derivatives are associated with various human pathologies, including diabetes, cancer, cardiac hypertrophy, and vitamin B12 deficiency (Reibel et al., 1981; McAllister et al., 1988; Brass et al., 1990). Inborn mutations in the human genes encoding two rate-limiting enzymes of the CoA biosynthetic pathway (*PANK2* and *COASY*) have been implicated in neurodegeneration with brain iron accumulation (NBIA) demonstrating the importance of CoA/CoA derivatives in the maintenance of central nervous system function (Zhou et al., 2001; Dusi et al., 2014).

A novel function of CoA in the antioxidant defense mechanisms has been recently revealed in our laboratory. Using cell-based and animal models, we demonstrated covalent modification of cellular proteins by CoA in cellular response to oxidative or metabolic stress (Tsuchiya et al., 2017, 2018). To discover and study this novel PTM termed “protein CoAlation,” we have developed novel reagents and methodologies: (a) anti-CoA monoclonal antibodies, which specifically recognize free CoA and CoA bound to proteins via a disulfide bond in ELISA, Western blotting, immunoprecipitation, immunohistochemistry; (b) a reliable mass spectrometry-based methodology for the identification of CoAlated proteins; and (c) efficient *in vitro* CoAlation and deCoAlation assays (Malanchuk et al., 2015; Tsuchiya et al., 2017, 2018). To date, over 2200 CoAlated proteins have been identified in prokaryotic and eukaryotic cells exposed to oxidative or metabolic stress. Protein CoAlation has been shown to regulate the activity and subcellular localization of modified proteins, protect oxidized cysteine residues from irreversible overoxidation, and to induce conformational changes (Gout, 2018, 2019; Bakovic et al., 2019; Tossounian et al., 2020; Tsuchiya et al., 2020; Yu et al., 2021). The antioxidant function of CoA and protein CoAlation in pathologies associated with oxidative stress, such as neurodegeneration, cancer, and diabetes, remains to be investigated.

Here, immunohistochemistry analysis with anti-CoA mAb was used to examine the extent of protein CoAlation in post-mortem human brain tissues from NBIA, AD, Corticobasal Degeneration (CBD), Progressive Supranuclear Palsy (PSP), Multiple System Atrophy (MSA), Parkinson’s Disease (PD), and matched controls. This analysis revealed positive immunoreactivity with anti-CoA in different structures within the brain tissue of NBIA, CDB, PD, and AD, when compared to matched controls. No anti-CoA immunoreactive signal was observed in PSP or MSA. Extensive anti-CoA immunoreactivity was detected in all brain regions apart from the basal ganglia in 70% of AD samples. Notably, the anti-CoA immunoreactive signal is readily observed in structures resembling NFTs. Double immunohistochemistry with anti-tau and anti-CoA antibodies showed co-localization of both antibodies within NFTs. This data encouraged us to demonstrate CoAlation of recombinant 2N4R and 2N3R tau isoforms, which was subsequently confirmed by mass spectrometry to occur at the conserved cysteine residue (Cys322 of 2N4R and Cys291 of 2N3R tau isoforms). Furthermore, reversible H_2_O_2_-induced dimerization of recombinant 2N3R, but not 2N4R isoform was reproducibly observed and shown to be completely inhibited by *in vitro* CoAlation. We have also found that transiently overexpressed tau is CoAlated in HEK2093/Pank1β cells treated with the thiol-oxidizing agent diamide. Considering the importance of cysteines in tau acetyl-transferase activity, we speculate that CoAlation of Cys322 would inhibit this function. Further, since the conserved cysteine residue is located within the microtubule binding region, tau CoAlation may potentially modulate its binding to microtubules and/or form a new binding site for regulatory interactions in redox signaling. Overall, CoAlation of tau may protect redox-sensitive cysteine 322 from irreversible overoxidation, modulate its acetyl-transferase activity and regulatory interactions.

## Materials and Methods

### Materials

All chemicals were purchased from Sigma–Aldrich unless otherwise noted here. Anti-CoA monoclonal antibody was produced via hybridoma cell line 1F10 (Malanchuk et al., 2015) and used in following dilutions (1:200 for IHC and 1:6,000 in WB). Biotinylated anti-mouse or anti-rabbit, respectively (both 1:200; Invitrogen). Monoclonal anti-tau (1:200 for IHC) was purchased from Thermo Fisher Scientific. All cell lines were purchased from American Tissue Culture Collection (ATCC, Manassas, VA, United States). Alexa Fluor 555- and 647-conjugated secondary antibodies (1:10,000 for WB) were purchased from Invitrogen.

Brains were donated to the Queen Square Brain Bank (QSBB) for neurological disorders (UCL Queen Square Institute of Neurology). All tissue samples were donated with the full, informed consent. Accompanying clinical and demographic data of all cases used in this study were stored electronically in compliance with the 1998 data protection act and are summarized in Table 1. Ethical approval for the study was obtained from the NHS research ethics committee (NEC) and in accordance with the human tissue authority’s (HTA’s) code of practice and standards under license number 12198.

**TABLE 1:** Summarized case demographic data for the post-mortem cases used in the study.

Case ID	AAO (years)	AAD (years)	DD (years)	Sex	Path diagnosis	PM delay (hours)	Brain weight (g)	Braak Tau	Thal	CERAD	ABC score
1	59	74	15	F	AD	102:55:00	1265	6	4	3	A3B3C3
2	59	76	17	F	AD	60:25:00	1191	6	5	3	A3B3C3
3	60	71	11	M	AD	52:39:00	1327	6	3	3	A2B3C3
4	74	79	5	F	AD	60:40:00	1200	6	5	3	A3B3C3
5	52	68	16	M	AD	73:45:00	1234	6	5	3	A3B3C3
6	55	67	12	M	AD	28:35:00	1015	6	5	3	A3B3C3
7	72	88	16	M	AD	58:10:00	1084	6	5	2	A3B3C2
8	63	73	10	M	AD	31:10:00	1269	6	5	3	A3B3C3
9	49	62	13	F	AD	76:40:00	996	6	5	3	A3B3C3
10	63	74	11	M	AD	33:26:00	1022	6	5	3	A3B3C3
11	48	61	13	M	AD	40:40:00	1650	6	5	3	A3B3C3
12	58	72	14	F	AD	81:26:00	820	6	5	3	A3B3C3
13	48	63	15	M	AD	31:42:00	1042	6	5	3	A3B3C3
14	51	69	18	F	AD	81:00:00	1056	6	5	3	A3B3C3
15	54	65	11	M	AD	34:25:00	1089	6	5	3	A3B3C3
16	na	103	na	F	Control	26:35:00	975	4	5	1	A3B2C1
17	na	88	na	M	Control	27:30:00	1330	4	3	2	A2B2C2
18	na	83	na	M	Control	105:28:00	1244	4	3	2	A2B2C2
19	na	92	na	M	Control	46:15:00	1213	4	3	2	A2B2C2
20	na	91	na	F	Control	69:20:00	1311	4	4	2	A3B2C2
21	60	68	8	F	PSP	36:50:00	1177	1	1	0	A1B1C0
22	75	84	9	F	PSP	70:00:00	1095	0	3	1	A2B0C1
23	76	84	8	M	PSP	50:00:00	1370	0	4	1	A3B0C1
24	57	62	5	M	PSP	72:20:00	1369	5	5	2	A3B3C2
25	66	79	13	F	PSP	73:55:00	1141	0	3	1	A2B0C1
26	58	65	7	M	CBD	48:04:00	1232	0	0	0	A0B0C0
27	54	61	7	M	CBD	102:30:00	1389	0	0	0	A0B0C0
28	58	69	11	F	CBD	103:15:00	917	0	0	0	A0B0C0
29	57	64	7	M	CBD	41:25:00	1456	0	0	0	A0B0C0
30	63	69	6	M	CBD	81:36:00	1291	0	2	1	A1B0C1
31	60	84	24	M	PD	71:05:00	1484	2	1	0	A1B1C0
32	80	89	9	M	PD	26:45:00	1493	4	4	2	A3B2C2
33	69	78	9	M	PD	95:15:00	1600	2	5	2	A3B1C2
34	77	83	6	M	PD	96:25:00	1644	1	3	1	A2B1C1
35	65	78	13	F	PD	84:50:00	1203	1	1	0	A1B1C0
36	67	75	8	M	MSA	54:00:00	1359	1	1	0	A1B1C0
37	46	52	6	F	MSA	79:00:00	1354	1	0	0	A0B1C0
38	33	42	9	M	MSA	30:10:00	1380	0	0	0	A0B0C0
39	63	72	9	M	MSA	82:00:00	1450	1	0	0	A0B1C0
40	57	63	6	M	MSA	102:55:00	1234	0	2	0	A1B0C0

*Detailing the number of cases used for each neurodegenerative disease; Alzheimer’s disease (AD), Progressive supranuclear palsy (PSP), coricobasal degeneration (CBD), Parkinson’s disease (PD), and Multiple system atrophy (MSA). Detailing the mean age at disease onset (AAO), mean age at death (AAD), disease duration (DD), mean hours to post-mortem (PM), and the brain weight.*

All cases were diagnosed pathologically according to current consensus criteria (Montine et al., 2012; Kovacs, 2015). The cohort included pathologically diagnosed cases of AD (*n* = 15), CBD (*n* = 5), PSP (*n* = 5), MSA (*n* = 5), PD (*n* = 5), and neurologically normal controls (*n* = 5). The control cases used in this study had no clinical symptoms of a neurodegenerative disease reports, however, they all had a certain degree of Aβ and tau deposition (Supplementary Table 1).

### Methods

#### Immunohistochemistry

A total of 8 μm paraffin-embedded formalin-preserved tissue sections were cut from the hippocampus and temporal cortex. Routine IHC was performed on sequential sections using anti-tau and anti-CoA, an in-house developed monoclonal antibody. In brief, tissue sections were de-waxed in xylene and rehydrated through various alcohol concentrations, then pre-blocked using methanol and hydrogen peroxide to prohibit endogenous peroxidase activity. Antigen retrieval was carried out by pressure cooking slides in pH 6.0 citrate buffer. A solution of 10% milk/TBS-T was used to prevent non-specific antigen/antibody binding. Tissue sections underwent incubation with primary antibody anti-CoA (1:200; inhouse) or anti-tau (1:200; Invitrogen) for 1 h at room temperature, followed by biotinylated anti-mouse secondary antibody (1:200; DAKO) and finally avidin-biotin complex (ABC), both for 30-min incubations. Di-aminobenzidine (DAB) was used as the chromogen. Sections were counterstained in Mayer’s hematoxylin, dehydrated, cleared, and mounted.

#### Double Staining Immunohistochemistry

After identical initial pre-treatments to routine IHC, tissue samples were similarly incubated with anti-CoA (1:200; inhouse), biotinylated anti-mouse (1:200; DAKO), and ABC. This was followed by 20 min incubation with Tyramide Signal Amplification (TSA) Red. Samples were then incubated with anti-tau polyclonal antibody (1:200; Invitrogen) and Alexa Fluor 488 Anti-Rabbit (1:1000; Invitrogen) for one and 2 h respectively at room temperature. 4′-6-diamidino-2-phenylindol (DAPI) was used to counterstain nuclei (1:1000; Vector) before slides were viewed under Leica confocal fluorescent microscope.

#### Quantitation of Neurofibrillary Tangles

The number of CoA and Tau positive NFTs were quantitated in the second frontal gyrus gray matter from the AD cases. IHC sequentially stained slides were scanned using an Olympus Slide Scanner at x20 magnification. Regions of interest were extracted from the digital images and the number of NFTs counted. The numbers of NFTs were then corrected for area (number of NFTs per mm^2^). The percentage of CoA positive NFTs were then calculated against the number of tau positive NFTS.

#### Purification of Recombinant 2N3R and 2N4R Tau Isoforms

Recombinant 2N4R and 2N3R tau isoforms were purified as described in Ferrari and Rüdiger (2018). Briefly, Rosetta (DE3) cells containing pSUMO-Flag 2N4R or 2N3R tau plasmid were grown in YT medium supplemented with 10 mg/L of kanamycin (Sigma-Aldrich) and 33 mg/L of chloramphenicol (Sigma-Aldrich), until OD_600_ reached 0.8. Subsequently, 2N4R and 2N3R tau expression was induced with 0.15 mM IPTG followed by incubation at 18°C for 16 h. Cells were then harvested, and the pellets were resuspended in lysis buffer (50 mM HEPES pH 8.5, 50 mM KCl, half a tablet of EDTA-free protease inhibitor (Roche), 5 mM β-mercaptoethanol). The cells were lysed and centrifuged, to remove cell debris. The lysates were loaded onto a POROS 20MC affinity purification column, with a column, and the proteins were eluted with a 0–100% gradient of 0.5 M imidazole. Fractions of interest were loaded onto a POROS 20HQ anion exchange column. The proteins were then eluted using a 0–100% linear gradient of 1 M KCl, which was then repeated using a POROS 20HS column for cation exchange of fractions of interest. The final fractions were concentrated (Vivaspin, cut-off 5 kDa), and stored at −20°C with DTT.

#### *In vitro* CoAlation of Recombinant 2N3R and 2N4R Tau Isoforms

Prior to the *in vitro* CoAlation assay of the 2N3R and 2N4R tau isoforms, the proteins were incubated for 30 min at room temperature with DTT (10 mM). Micro Biospin 6 columns (BioRad), equilibrated with 1X PBS, were used to remove excess of DTT. 2N3R and 2N4R tau isoforms (8 μM) were incubated (40 min, 25°C) with H_2_O_2_ (200 μM) in the presence or absence of CoA (70 μM). To stop the reaction, 5 mM N-ethyl maleimide (NEM) was added, and the samples were incubated for 10 min at 25°C. NEM is a thiol alkylating agent.

To study the reversibility of tau CoAlation or dimerization, two additional samples were prepared. Following oxidation and/or CoAlated of the 2N3R and 2N4R tau isoforms, 5 mM DTT (reducing agent) was added to each sample. The samples were then incubated for 10 min at 25°C. The reaction was stopped by the addition of NEM. Following alkylation with NEM, the samples were boiled in 1X non-reducing loading buffer and separated by SDS-PAGE.

#### Anti-CoA Western Blot Analysis of CoAlated 2N3R and 2N4R Tau Isoforms

Resolved proteins were transferred to low-fluorescence polyvinylidene fluoride (PVDF) membranes (BioRad) according to manufacturer’s instruction. Membranes were blocked in LiCor blocking buffer for 30 min at room temperature (RT), then washed three-times for 3 min in 1X Tris-buffered saline with 0.05% tween-20 (TBS-T). The membranes were then incubated overnight at 4°C with mouse anti-CoA monoclonal antibodies (mAbs) (1:6,000). The membranes were then washed three-times for 3 min with 1X TBS-T, and incubated for 30 min at RT with goat anti-mouse AlexaFluor680 antibodies (1:10,000). Washing was repeated three-times for 3 min in 1X TBS-T then again in 1X TBS. Fluorescence signal at 702 nm was measured using LiCor Odyssey-CLx.

#### Liquid Chromatography-Tandem Mass Spectrometry Analysis of CoAlated 2N3R and 2N4R Tau Isoforms

Reduced 2N3R and 2N4R tau isoforms (10 μM) were incubated for 30 min at 25°C with H_2_O_2_ (100 μM) in the presence of CoA (100 μM). To stop the reaction, 5 mM N-ethyl maleimide (NEM) was added, and the samples were further incubated for 10 min at 25°C. Excess CoA, H_2_O_2_ and NEM were removed by desalting using the MicroBiospin6 columns. The samples were then analyzed by MS.

The LC-MS/MS analysis of CoAlated peptides was carried out as described previously (Brass et al., 1990; Dusi et al., 2014). Briefly, the samples predicted to contain CoAlated tau were tryptic digested, and the peptides were analyzed by nano-scale capillary liquid chromatography-tandem mass spectrometry (LC-MS/MS) using an Ultimate 3000 RSLC System (Thermo Fisher Scientific) integrated with a 100 μm × 2 cm PepMap100 C_18_ nano-trap column and an EASY-Spray PepMap RSLC C_18_ 2 μm, 25 cm × 75 μm analytical column (Thermo Fisher Scientific). Nano-flow electrospray ionization was used to directly spray peptides eluted by an acetonitrile gradient into the Q Exactive Orbitrap mass spectrometer (Thermo Fisher Scientific). Operated in data-dependent mode, the mass spectrometer utilized an Orbitrap analyzer with a resolution of 35,000 at mass-to-charge ratio (m/z) of 200. Subsequently, MS/MS of the 10 most intense ions were acquired. Maximum accumulation time for MS full scan and MS2 were set at 50 and 100 ms, respectively. Internal calibration of Orbitrap measurements involved the lock mass of polydimethylcyclosiloxane at m/z 445.120025. MaxQuant v1.6.6.0 was used to process LC-MS/MS raw data files (Cox and Mann, 2008). Processed data were searched against Human (2019) UniProt protein databases^1^. Carbamidomethyl cysteine, Acetyl N-terminal, N-ethylmaleimide cysteine, oxidation of methionines, and CoAlation of cysteine with delta mass 356 and 765, were set as variable modifications. All data sets used default MaxQuant parameters with the second peptide ID remaining unselected.

#### Generation of the pEGFP-N1/His-2N4R Plasmid

6xHis-tag sequence was introduced at the N-terminus of WT 2N4R *tau* (pEGFP-N1 plasmid) to generate the His-2N4R tau-pEGFP-N1 plasmid. Using phosphorylated Fw (5′-ATGGGCAGCCAT CATCATCATCATCACAGCGGCATGGCTGAGCCCCGCCA G-3′) and Rv (5′-GGTGGCAGA TCTGAGTCCGGTAGC-3′) primers, the plasmid was amplified and an overhang with the His-tag sequence was introduced. T4 DNA ligase (Thermo Fisher Scientific) was used to circularize the plasmid, which was then electroporated in *E. coli* Top10 cells. The plasmid was then amplified and purified. The integration of the 6xHis-tag at the N-terminus of the 2N4R *tau* within the pEGFP-N1 plasmid was confirmed by DNA sequencing. The presence of a stop codon at the C-terminus of 2N4R *tau* prevents the expression of downstream GFP sequence in the pEGFP-N1 plasmid.

#### Mammalian Cell Culturing, Transfection, and Treatment With Diamide

In this study, we used HEK293 cells with stable overexpression of Pank1β. Overexpression of this rate-limiting enzyme in CoA biosynthesis increases the level of CoA to that observed in primary cell lines (rat primary cardiomyocytes) and rat tissues (liver, heart, or kidney) (Tsuchiya et al., 2017). The increase in CoA level in HEK293/Pank1β cells results in significant increase of protein CoAlation in response to oxidative or metabolic stress (Tsuchiya et al., 2017, 2020; Bakovic et al., 2019; Yu et al., 2021). HEK293/Pank1β cells were maintained in DMEM (Dulbecco’s Modified Eagle Medium) supplemented with fetal bovine serum (10% – FBS, Gibco), penicillin (50 U/mL) and streptomycin (0.25 μg/mL – Lonza) at 37°C and 5% CO_2_. Around 0.6 million cells were seeded onto 60 mm plates, and at ∼60% confluency, the cells were transiently transfected with pEGFP-N1/His-2N4R *tau* plasmid using XtremeGene HP transfection reagent (Roche), according to the manufacturer’s protocol. After 24 h, cells were primed for oxidative stress by changing the medium to pyruvate and glucose-free DMEM (10% FBS) supplemented with 5 mM glucose. After 18 h of culturing, cells were treated with diamide (500 μM) for 30 min at 37°C. After harvesting, cells were lysed on ice for 20 min in lysis buffer [50 mM Tris pH 7.5, 150 mM NaCl, 5 mM EDTA, 50 mM NaF, and 5 mM sodium pyrophosphate, 1% triton X-100, 1X PIC (cOmplete mini protease inhibitor cocktail), and 25 mM NEM]. Following centrifugation, 30 μg of lysate was mixed/boiled with SDS-PAGE loading dye, and the rest of the lysate was incubated overnight at 4°C with nickel-NTA beads equilibrated in wash buffer (lysis buffer without PIC and NEM). The beads were then washed 3 times with lysis buffer and mixed/boiled with SDS-PAGE loading dye. Immunoblotting with mouse anti-CoA (1:6,000) and mouse anti-tau12 (Merck) (1:5,000) was performed as described earlier.

## Results

### Optimization of Anti-CoA Immunohistochemistry

Recent discovery of the antioxidant function of CoA and the development of anti-CoA monoclonal antibody 1F10, which works efficiently in various immunological approaches (Malanchuk et al., 2015), prompted us to investigate its suitability for immunohistochemical (IHC) analysis of post-mortem human tissues. In previous studies, we showed that 1F10 antibody specifically recognizes in Western blotting CoA covalently bound to proteins via a disulfide bond in mammalian cells and tissues, exposed to oxidative or metabolic stress (Tsuchiya et al., 2017, 2018). Moreover, this antibody is also efficient in immunoprecipitating CoAlated peptides, when employed for the identification of CoA-modified proteins by mass spectrometry (Malanchuk et al., 2015).

In this study, our efforts were focused on examining the suitability of 1F10 antibody for IHC analysis of protein CoAlation in pathologies associated with oxidative stress. Initial optimization studies of 1F10 antibody were carried out in post-mortem NBIA brain samples and age-matched controls with no cognitive impairment. Several pre-treatments were tested (no pre-treatment, pressure cooking in citrate buffer pH6.0, proteinase K and formic acid) to determine the best pre-treatment and this was paired with titrating the antibodies concentration for optimum staining. We determined that pre-treating the section in a pressure cooker and using the antibody at 1:200 ratio, gave the optimum staining intensity compared to background staining in formalin fixed paraffin embedded tissue.

**FIGURE 1:**
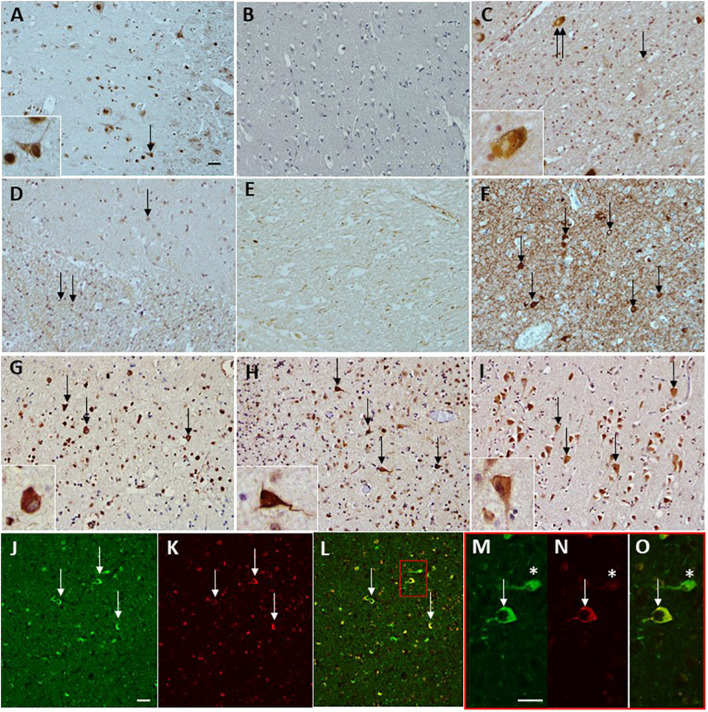
Immunohistochemical staining of anti-CoA and tau antibodies in neurodegenerative diseases. CoA deposition is found in a case with NBIA **(A)** used as a positive control for the immunohistochemical preparations. No CoA immunoreactivity was observed in a neurologically normal control without the presence of tau pathology **(B)**. CoA immunoreactivity was observed in PD, in the occasional Lewy body (**C**, double arrow and insert), as well as the occasional Lewy neurite (**C**, arrow). No CoA immunoreactivity was observed in either MSA white matter (WM) or gray matter (GM). Both PSP and CBS were negative for CoA in the frontal and temporal cortices. However, axonal CoA staining was observed in the basal ganglia in CBD **(E)**. Tau immunohistochemistry demonstrating the level of tau pathology in the form of neurofibrillary tangles (**F**, arrows) surrounded by neuropil threads in an AD case. CoA immunohistochemistry in AD also shows positivity in the neurofibrillary tangles in the frontal cortex (**G**, arrows and insert), temporal cortex (**H**, arrows and insert), and CA1 subregion of the hippocampus (**I**, arrows and insert). Double immunohistochemical staining with CoA (red; **K,N**) and tau (green; **J,M**) shows co-localization of the two proteins (arrows, **L**) and at higher magnification **(O)**, but also reveals neurofibrillary tangles that are negative for CoA (asterisks, **M–O**). Bar in a represents 50 μm in panels **(A–L)**, inserts 20 μm. Bar in panels **(J,M)** represents 50 μm. PD, Parkinson’s disease; MSA, multiple system atrophy; AD, Alzheimer’s disease; PSP, progressive supranucelar palsy; CBD, corticobasal degeneration.

Using optimized IHC conditions (Figure 1), we observed readily detectable anti-CoA immunoreactive signal in NBIA brain samples located predominantly in the neuronal and glial nuclei, as well as cytoplasmic staining in the larger neurons in the gray matter (Figure 1A and insert). In contrast, anti-CoA immunoreactivity of age-matched controls demonstrated very little nuclear staining (Figure 1B).

### Anti-CoA Immunohistochemistry of Post-mortem Brain Tissues of Patients With Various Neurodegenerative Pathologies

Following the optimization studies and the positive IHC staining found in the NBIA brain samples, we investigated the profile of anti-CoA immunoreactivity in brain tissues from several neurodegenerative diseases and age-matched controls. These included AD, Corticobasal Degeneration (CBD), Progressive Supranuclear Palsy (PSP), Multiple System Atrophy (MSA), and Parkinson’s disease (PD). Brain regions where the majority of pathology is exhibited in each disease were analyzed, including frontal cortex, temporal cortex, hippocampus, basal ganglia, upper midbrain, and anterior cingulate.

The IHC analysis showed that other neurodegenerative diseases exhibited varying degrees of positive immunoreactivity with anti-CoA, which was typically seen in different pathological structures within the brain tissue. In a small subset of PD samples (15%), positive anti-CoA staining of structures resembling Lewy body (Figure 1C and insert) were present in the anterior cingulate and upper midbrain. No anti-CoA immunoreactivity was observed in either gray or white matter in MSA (Figure 1D) or in any of the PSP cases. In 20% of CBD samples, anti-CoA immunoreactive staining was primarily observed in the neuronal axons in the white matter of basal ganglia samples (Figure 1E).

Anti-CoA immunoreactive signal was observed in AD brain samples (frontal cortex) in structures resembling neuropil threads and NFT’s (Figures 1G–I, arrows).

### Analysis of Anti-CoA Immunoreactivity in Alzheimer’s Disease Brain Samples

The anti-CoA immunoreactivity was detected in all brain regions, apart from the basal ganglia, in AD brain samples. When compared to control samples (Figure 1B), anti-CoA immunoreactivity in AD brain samples was detected consistently in assemblies resembling NFT’s and neuropil threads, both of which are characteristic pathophysiological markers of AD (Figure 1F). The immunoreactive signal was observed in frontal cortex (Figure 1G), temporal cortex (Figure 1H), and hippocampus (Figure 1I) but not in the basal ganglia. Taking into account that the structures stained with anti-CoA antibody resembled tau-positive NFT’s, the same cases and brain regions were analyzed using anti-tau antibody (Figure 1F) and showed strong immunoreactivity, including in structures similar to NFT.

**FIGURE 2:**
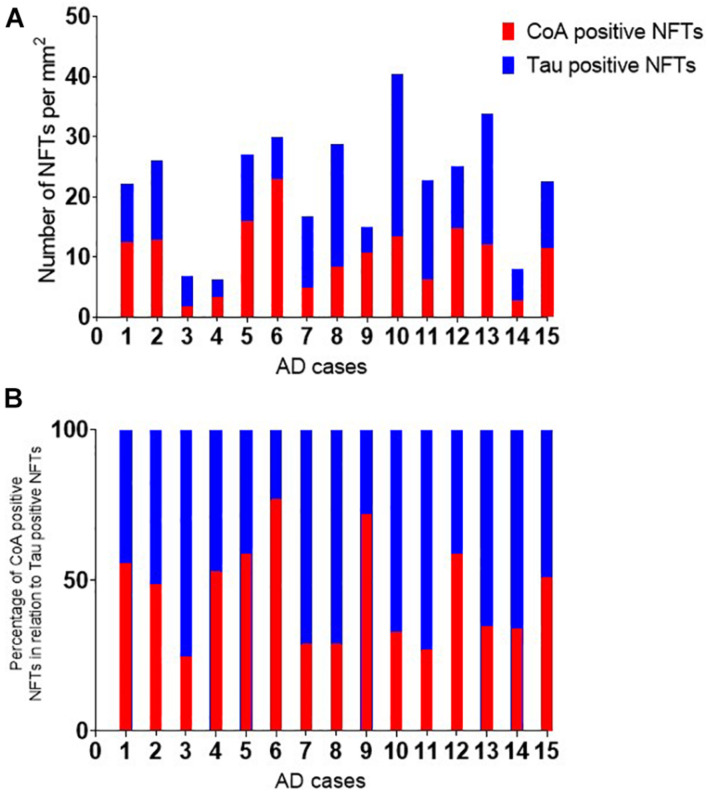
Quantitative analysis of Tau and CoA positive neurofibrillary tangles. The number of neurofibrillary tangles (NFTs) positive with tau and CoA immunohistochemistry were quantitated in the frontal cortex of Alzheimer’s disease cases. The number of NFTs were counted in a region of interest in the frontal gray matter and corrected per mm^2^. **(A)** The percentage of CoA NFTs relative to the number of tau positive NFTs are shown in panel **(B)**.

To investigate whether anti-CoA and anti-tau immunoreactive signals co-localize in NFTs, double staining IHC of AD brain samples with tau and CoA antibodies was carried out. Immunoreactivity with anti-CoA (Figures 1K,N) and anti-tau (Figures 1J,M) was clearly observed and immunofluorescent co-localization of the two antibodies within NFT structures was revealed, demonstrated by the yellow immunofluorescent signals (Figures 1L,O). Notably, antibody co-localization was not seen with the same intensity at every instance where anti-tau had bound to NFT proteins (Figures 1M–O, asterisks), suggesting CoAlation of NFTs is not uniform or an inevitable PTM in these structures. Quantitative analysis was undertaken to assess the number of CoA positive NFTs compared to the number of tau positive NFTs (Figures 2A,B). NFTs were counted in the frontal gray matter. In all AD cases a large proportion of NFTs were found to be CoA positive. The average number of CoA positive NFTs per mm^2^ was 10 and the average number of Tau positive NFTs was 22 per mm^2^, equating on average to around 50% of tau positive NFTs were also CoA positive.

### 2N3R and 2N4R Tau Isoforms Are CoAlated *in vitro*

**FIGURE 3:**
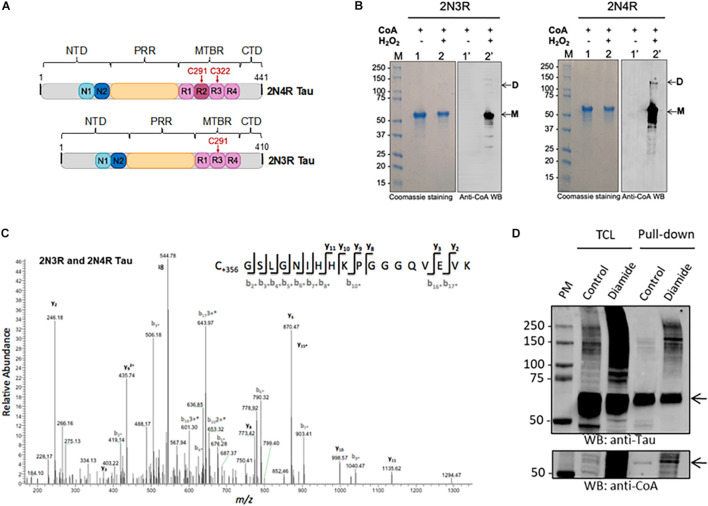
Analysis of tau CoAlation *in vitro* and *in vivo*. **(A)** Schematic diagram of human 2N4R and 2N3R tau isoforms (441 and 410 amino acids in length, respectively). N-terminal inserts, proline rich region (PRR), and microtubule-binding region (MTBR) are shown in blue, yellow, and pink, respectively. Conserved cysteine residues within MTBR are indicated in red. 2N3R tau lacks the second MTBR and thus only features the conserved cysteine residue (in this case, Cys291). N- and C-terminal domains (NTD and CTD, respectively) are shown in gray. **(B)** *In vitro* CoAlation of recombinant 2N4R and 2N3R tau isoforms occurs following exposure to hydrogen peroxide. Lane L indicates protein molecular weight marker. Lanes 1 and 1′ contain reduced tau in the presence of CoA. Lanes 2 and 2′ contain H_2_O_2_- and CoA-treated tau. CoAlated tau remains largely monomeric (M) yet CoAlated dimers are also present for 2N4R **(D)**. **(C)** Mass spectrometry analysis of the CoAlated peptide (C*GSLGNIHHKPGGGQVEVK) indicates covalent modification of cysteine (C_+__356_) by CoA at the conserved cysteine residue (Cys322 and Cys291 in 4R and 3R tau, respectively). **(D)** Transiently overexpressed His-2N4R tau is CoAlated in HEK293/Pank1β cells exposed to diamide-induced oxidative stress. Total cell lysates and Ni-NTA pulled-down proteins were separated by SDS-PAGE under non-reducing conditions and immunoblotted with anti-CoA and anti-tau12 antibodies. The position of His-2N4R tau in total lysates and pulled-down samples is shown with the arrow. Oligomerization of His-2N4R tau in total lysates and pulled-down samples readily detected with anti-Tau antibodies. TLC, total cell lysate; Pull-down, the use of Ni-NTA affinity beads to pull-down His-tag fusion proteins from cell lysates.

In this study, we used 2N3R and 2N4R tau isoforms, which are distinguished only by the configuration of their microtubule-binding repeat domains. Within the second and third microtubule-binding repeat domains (R2 and R3, respectively) are one or two naturally occurring cysteine residues (according to nomenclature of the longest tau isoform, Cys291 and Cys322, respectively) (Figure 3A). 2N3R tau does not possess the second repeat domain (R2) and therefore only contains the cysteine within R3, which is at position 291. 2N3R Cys291 and 2N4R Cys322 are therefore structurally equivalent and highly conserved (Figure 3A).

We expressed and purified 2N3R and 2N4R tau proteins, which were used in the *in vitro* CoAlation assays. The *in vitro* tau CoAlation reaction was carried out in the presence of H_2_O_2_ and CoA, and the samples were then separated by SDS-PAGE under non-reducing conditions and immunoblotted with anti-CoA 1F10 antibody. Both 2N3R and 2N4R tau proteins were found to be efficiently CoAlated *in vitro* (Figure 3B). Both isoforms were predominantly CoAlated in the monomeric state, but low level of CoA-modified 2N4R dimer was also detected. Mass spectrometry analysis of CoAlated samples revealed one CoA-modified peptide (C^∗^GSLGNIHHKPGGGQVEVK) in both 2N3R and 2N4R isoforms (Figure 3C). Cys322 was CoAlated in 2N4R isoform, and Cys291 in the 2N3R isoform. Multiple sequence analysis of 2N3R and 2N4R tau isoforms (data not shown) revealed this CoAlated cysteine residue to be the ubiquitously conserved cysteine across all isoforms of tau, and present within R2 and R3 of 2N3R and 2N4R tau, respectively.

### Diamide-Induced Oxidative Stress Causes CoAlation of His-2N4R Tau in HEK293/Pank1β Cells

To explore the role of tau CoAlation *in vivo*, HEK293/Pank1β cells transiently over-expressing His-2N4R tau were incubated with or without diamide as described in M&M. His-2N4R tau was pulled-down from lysed cells using Nickel-NTA beads. The pulled-down protein was then mixed/boiled with SDS-PAGE loading dye, and separated on SDS-PAGE gel under non-reducing conditions. Anti-tau12 and anti-CoA Western blots were performed to visualize the expression and pull-down efficiency of His-2N4R tau, and the pattern of CoAlation in analyzed samples. As shown in Figure 3D, His-2N4R tau is efficiently expressed in HEK293/Pank1β cells and pulled-down on Nickel-NTA beads. We have reproducibly observed CoAlation of His-2N4R tau in samples prepared from diamide-treated HEK293/Pank1β cells. Interestingly, a significant increase in tau oligomerization was detected in cells exposed to diamide, when compared to non-treated controls.

### Reversible *in vitro* CoAlation of 2N3R Prevents H_2_O_2_-Induced Tau Dimerization

**FIGURE 4:**
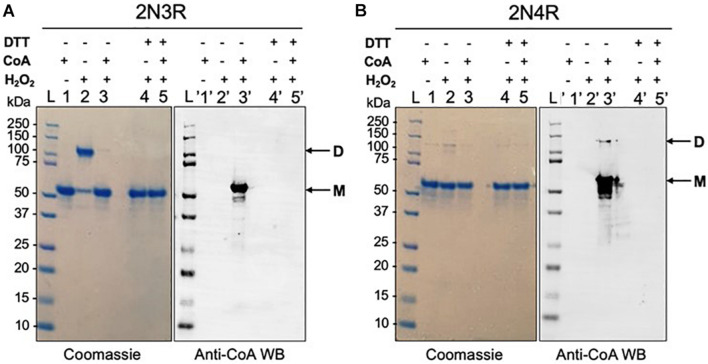
H_2_O_2_-induced dimerization of 2N3R tau isoform is inhibited by CoAlation. **(A)** H_2_O_2_-treatment of 2N3R tau causes its dimerization (Lane 2 – D). In the presence of CoA and H_2_O_2_, 2N3R becomes *in vitro* CoAlated (Lane 3′), and its dimerization is inhibited (Lane 3 – M). In the presence of DTT, both dimerization and CoAlation of 2N3R tau are inhibited (Lanes 4, 4′, 5 and 5′ – M). **(B)** H_2_O_2_-treatment of 2N4R tau shows only a minor dimerization band (Lane 2and 2′ – M and D), probably due to the formation of intramolecular disulfide bond formation, rather than an intermolecular disulfide formation. H_2_O_2_ and CoA-treated 2N4R tau shows CoAlation (Lane 3′ – M and D). In the presence of DTT, CoAlation is absent (Lane 5′). Lane L represents the molecular weight marker, and lane 1 represents the reduced form of 2N3R and 2N4R tau isoforms in the presence of CoA, where CoAlation is not observed (Lane 1′).

To further understand the role of CoA and tau CoAlation in the presence of H_2_O_2_, which is a strong oxidant, we analyzed the oligomerization and CoAlation states of the H_2_O_2_-treated 2N3R and 2N4R tau isoforms in the presence and absence of CoA (Figures 4A,B). H_2_O_2_-induced dimerization of the 2N3R tau isoform is reproducibly observed and mediated via the formation of an intermolecular disulfide bond (Figure 4A – Sample 2). On the other hand, 2N4R tau isoform, showed a weaker dimerization band (Figure 4B – Sample 2), as it may be more likely to engage in intramolecular disulfide bond formation due to the proximity of the two cysteine residues (Cys291 and Cys322) within the MTBR.

To determine whether CoAlation of the 2N3R tau isoform interferes with its dimerization, the protein was incubated with H_2_O_2_ in the presence of CoA. Interestingly, H_2_O_2_-induced 2N3R dimerization is nearly completely inhibited in the presence of CoA (Figure 4A – Sample 3). With anti-CoA WB, we show that the latter monomeric band of 2N3R tau is strongly CoAlated (Figure 4A – Samples 3 and 3′). This may indicate that CoAlation protects 2N3R tau from disulfide bond-mediated dimerization. For the 2N4R tau isoform, both the monomeric (major form) and dimeric (minor form) bands showed CoAlation in the presence of H_2_O_2_ and CoA (Figure 4B – Sample 3′). As the dimeric 2N4R tau contains free thiols, CoAlation could occur on these residues. Overall, we showed that both 2N3R and 2N4R tau isoforms are CoAlated in the presence of H_2_O_2_, but 2N3R tau dimerizes more readily compared to 2N4R isoform. These findings suggest that CoA may have a protective role against H_2_O_2_-induced overoxidation of cysteine residues in tau.

To determine whether CoAlation of 2N3R and 2N4R tau isoforms in the presence of H_2_O_2_ is a reversible modification, CoAlated proteins were treated with excess of DTT, which is a reducing agent. For the case of the H_2_O_2_-treated 2N3R tau isoform, the dimerization band was completely shifted to its monomeric form (Figure 4A – Sample 4). DeCoAlation of 2N3R and 2N4R isoforms was also observed in the presence of DTT (Figures 4A,B – Samples 5 and 5′). Overall, these results suggest that CoAlation has a potential to protect the cysteine residues of tau from oxidative damage and the formation of intermolecular disulfide bond-mediated dimerization.

## Discussion

The antioxidant function of CoA and protein CoAlation have recently emerged as a novel mechanism of redox regulation in health and disease. The development of highly specific anti-CoA mAb and a reliable MS-based methodology allowed the identification of over 1400 CoAlated proteins in mammalian cells and tissues exposed to oxidative or metabolic stress. In this study, we report that anti-CoA immunoreactivity is readily observed by IHC in several neurodegenerative diseases, including NBIA and Parkinson’s disease, but with a substantial prevalence in AD. Utilizing anti-CoA mAb for the first time on post-mortem formalin fixed tissue, extensive protein CoAlation was detected in tau positive pathological structures in several brain regions in AD in contrast to control brain samples. Double immunohistochemical staining with tau and CoA antibodies in AD brain tissue revealed co-localization of the two immunoreactive signals in a proportion of NFTs, suggesting that protein CoAlation takes place during the maturation of the NFTs. We have also shown that recombinant 2N3R and 2N4R tau isoforms are CoAlated *in vitro* and the site of CoAlation mapped by mass spectrometry to the conserved cysteine residue among the tau isoforms. Furthermore, H_2_O_2_-induced oxidation can cause the dimerization of 2N3R, which was inhibited in the presence of both H_2_O_2_ and CoA. This could suggest a protective role of CoA against H_2_O_2_-induced oxidation and dimerization of tau. Therefore, we propose that covalent tau modification by CoA has a potential to protect tau cysteine residues from irreversible overoxidation.

The identification of CoAlation in NFTs raises more questions for extensive further studies. One key aspect to be addressed is that the double IHC staining revealed that CoA did not co-localize with every NFT. Speculation for the consequences of tau CoAlation can be drawn from what is known about existing PTMs that target and regulate this protein. Tau undergoes a plethora of modifications that are critical to its regulation and localization in AD brains, including – but not limited to – polyubiquitination, O-glycosylation, glycation, oxidation and phosphorylation, the latter of which has been most extensively reported (Gong et al., 2005). Indeed, in AD hyperphosphorylation of tau confers profound biological consequences. Tau contains an abundance of potential phosphorylation sites, including 80 serines/threonines and 5 tyrosines (Vasili et al., 2019). The addition of phosphoryl groups at these sites contributes to the loss of physiological tau function and its gain in toxicity as it self- assembles into PHFs mixed with straight filaments, aggregating into NFTs (Iqbal et al., 2010; Miao et al., 2019). It has been shown *in vitro* that prolong oxidative stress can increase tau phosphorylation (Martin et al., 2011). Therefore, it seems logical to postulate that during oxidative stress, the level of tau CoAlation would simultaneously increase. Existing research on tau oxidation is limited, but the assembly of PHFs *in vitro* and *in vivo* was found to be promoted by the oxidation of tau. In the largest human tau isoform (2N4R) oxidation has been reported at cysteine 322, which is localized in the R3 repeat domain of the microtubule binding domain region (Martin et al., 2011). Given the location of the cysteine residue in the microtubule binding domain, it could be plausible that the covalent attachment of CoA causes disruption to the association of tau to microtubules, diminishing its stabilizing effects and contributing to AD etiopathogenesis (as is seen in tau phosphorylation). CoA is a 767 Da molecule with a bulky 3′, 5′-ADP and pantetheine moieties, which may sterically hinder the interaction of CoA-modified tau with microtubules and promote regulatory interactions with proteins possessing the nucleotide binding fold. Targeted biochemical studies will be necessary to examine the consequences of CoAlation on binding properties and physiological functions of tau.

A hallmark occurrence of multiple incurable neurodegenerative diseases is abnormal protein aggregation and accumulation, including several clinicopathological entities in which tau aggregates are implicated, known as tauopathies including AD. Leading to cellular dysfunction, synaptic damage and neuronal death, aberrant protein aggregation in the brain is one of the main events in brain disease pathology (Soto, 2003; Ross and Poirier, 2004; Goedert, 2015). Tau is an IDP subject to highly sophisticated regulation by a diverse set of PTMs. Evidently, this has presented difficulties in the precise elucidation of the role of tau in AD and its position along the chain of events leading to neurodegeneration. Given the extensive pathological role of tau in numerous neurodegenerative diseases, the acceleration in the number of AD cases accompanying a continual expansion in life expectancy has created an indispensable requirement for AD therapeutic interventions. Despite substantial efforts, only symptomatic treatments exist for AD, and disease-modifying treatment strategies are still under extensive research (Yiannopoulou and Papageorgiou, 2020). To inhibit the progression of AD, treatments must interfere with pathogenic advancements responsible for clinical AD symptoms, including NFT formation and oxidative damage. Two types of tau filaments have been identified to be involved in the pathogenesis of AD. Together, PHFs and SFs comprise intraneuronal NFTs, a pivotal hallmark lesion of AD. Importantly, compelling evidence has shown that oxidation of tau cysteine in R3 is critical in NFT formation *in vitro* through the formation of intermolecular disulfide bridges formed by Cys322 (Schweers et al., 1995). This highlights the potential importance of oxidative stress tau aggregation, where the link between oxidation and AD is increasingly recognized. While the source of oxidative stress in AD is somewhat unclear, our data indicates that dimerization of tau induced by oxidizing agents could be a key step in tau aggregation. Certainly, this requires further investigation into the precise role of oxidative stress mechanisms in AD pathogenesis, and could lead to the development of novel clinical interventions acting to prevent tau dimerization via cysteines.

It is now well established that the brain is particularly vulnerable to oxidative stress due to high oxidative metabolic activity and lower level of antioxidants when compared to other tissues. The emerging antioxidant function of CoA mediated by protein CoAlation provides an additional mechanism for protecting surface exposed cysteines in neuronal proteins from overoxidation, including Cys322 in tau. Taking into account the involvement of tau cysteine residues in facilitating intrinsic acetyl-transferase activity, CoAlation of Cys322 under oxidative stress may mediate reversible inhibition and downstream effect of tau auto-acetylation.

Protein CoAlation has been shown to regulate the activity of modified proteins, in a manner similar to the regulatory changes induced by other covalent PTMs, including protein *S*-glutathionylation, phosphorylation, or acetylation. For instance, *S*-glutathionylated tau has been detected through mass spectrometry, and its function has been shown to alter the polymerization of 3-repeat tau (Landino et al., 2004). *S*-Glutathionylated 3-repeat tau was demonstrated to be capable of polymerization, which suggests that disulfide-linked tau dimerization is not an essential step in tau filament assembly leading to NFT formation. Further, recent studies have demonstrated double-cysteine tau mutants to be aggregation-competent. However, aggregation has been found to be dependent on cysteines for rapid initiation (Chidambaram and Chinnathambi, 2020). This suggests a significant role for disulfide bond formation in the early stages of tau aggregation, which may subsequently proceed via cysteine-independent mechanisms including hexapeptide motifs. Therefore, we hypothesize that the CoA antioxidant function could prevent intermolecular disulfide bond formation prompting accelerated tau aggregation during oxidizing conditions.

While our results primarily suggest a role of CoA in protecting tau from dimerization, which may lead its pathological accumulation, alternate mechanisms for tau aggregation exist. For instance, two hexapeptide motifs within the MTBR of tau are reported to be critical in driving tau fibril formation via the adoption of β-sheet structures (Von Bergen et al., 2000). Under physiological conditions tau protein is involved in the regulation of assembly, dynamic behavior, and the spatial organization of microtubules. Further, a model of tau-tubulin interaction suggests the formation of disulfide linkages between tau and tubulin cysteines: specifically, Cys291 of tau R2 first binds Cys347 of α-tubulin at “Site 1,” which allows Cys322 of tau R3 to subsequently bind C131 of β-tubulin at “Site 2” (Martinho et al., 2018). Therefore, we propose CoAlation at the conserved cysteine residue (Cys322 in 2N4R tau) could modulate disulfide bond mediated tau dimerization alongside the binding to tubulin. As the residues responsible for binding are located within the MTBR, this suggests that tau CoAlation may be involved in regulating microtubule-binding in response to fluctuations in the redox state of the cell. For instance, the presence of a bulky CoA group within the MTBR could sterically hinder access to the microtubule and inhibit the tau-tubulin functional interaction. As such, CoAlated tau would remain unable to bind microtubules for the duration of increased oxidative stress levels. When cells recover, deCoAlation of tau would allow microtubule stabilization to resume, thus proving an additional layer of regulation of cytoskeletal dynamics in response to cellular redox shifts.

Finally, it will also be interesting to consider the effect of tau CoAlation on other PTMs of tau. Importantly, tau is subject to regulation by a wealth of PTMs. Phosphorylation presents one of the most extensively studied PTMs of tau. Tau possesses over 80 potential sites for phosphorylation (Goedert et al., 1989). In AD, at least 40 phosphorylation sites have been identified in pathological tau through comprehensive MS analysis (Morishima-Kawashima et al., 1995; Hanger et al., 2007). However, a recent study (Wesseling et al., 2020) compiling an extensive catalog of PTMs including (but not limited to) ubiquitination, acetylation, and methylation across the full length of tau has highlighted the heterogeneity of these modifications. By nature, PTMs are incredibly complex as they occur in multitudes and may be combined in several ways. Tau PTMs are also prone to crosstalk and competition. For instance, acetylation of tau can also inhibit the phosphorylation of nearby residues. Acetylation of Lys321, Lys259, and Lys353 inhibits phosphorylation of Ser324, Ser262, and Ser356 respectively. This crosstalk generates complexity since acetylation is directly impairing but also indirectly activating tau functionality. A potential opposition in which the addition of certain chemical groups can block the addition of another on a given residue further suggests multifaceted regulation of tau biology (Liu et al., 2004, 2009; Yang and Seto, 2008; Bourré et al., 2018). As such, the recent explosion of knowledge on tau PTMs provides the opportunity to better understand these modifications in the context of tau homeostasis, and their perturbation in aging and disease. Oxidative stress was shown to induce the formation of the CoA biosynthetic complex and CoA production, a local increase in CoA occurs during protein acetylation, as acetyl-CoA provides the acetyl group for histone acetyl-transferases. The availability of reduced CoA in close vicinity may Therefore, it will be interesting to consider the effect of CoAlation on PTM of sites in tau in particular, phosphorylation and/or acetylation.

Overall, the involvement of the altered mitochondrial function and oxidative stress in neurodegenerative diseases including AD, combined with findings which link CoA dysregulation to neurodegeneration in NBIA raises the questions as to whether protein CoAlation could occur in neurodegenerative disorders and whether it has a protective role against oxidative damage and aggregation. In this study, we examined the pattern and distribution of CoAlation among different brain regions. We further explored tau CoAlation using *in vitro* studies, where we identified the site of tau CoAlation using MS, and determined that CoAlation can protect 2N3R tau from dimerization in the presence of H_2_O_2_.

## Data Availability Statement

The data presented in the study are stored electronically at the main UCL server in suitable file formats and will be available if requested by other researchers. The summary of case demographic data for the post-mortem cases used in the study is presented in Table 1.

## Ethics Statement

The studies involving human participants were reviewed and approved by the NHS Research Ethics Committee (NEC) and in accordance with the Human Tissue Authority’s (HTA’s) code of practice and standards under license number 12198. The patients/participants provided their written informed consent to participate in this study.

## Author Contributions

IG conceived the present study. TL, M-AT, and IG designed the experiments. TL and NC performed the IHC studies. M-AT performed the *in vitro* studies. M-AT, JC, AB, and SW performed the *in vivo* experiments. SP-C and MS performed the MS analysis. OM and VF developed and produced the anti-CoA monoclonal antibody. MK and SR purified the tau isoforms for the *in vitro* studies. TL, M-AT, NC, SW, AB, JC, RS, SS, MK, SR, and IG analyzed and discussed generated results. TL, M-AT, SW, and IG wrote the manuscript with the assistance and approval of all other authors. All authors contributed to the article and approved the submitted version.

## Conflict of Interest

The authors declare that the research was conducted in the absence of any commercial or financial relationships that could be construed as a potential conflict of interest.

## Publisher’s Note

All claims expressed in this article are solely those of the authors and do not necessarily represent those of their affiliated organizations, or those of the publisher, the editors and the reviewers. Any product that may be evaluated in this article, or claim that may be made by its manufacturer, is not guaranteed or endorsed by the publisher.

## Footnotes

1
https://www.uniprot.org/uniprot/

## References

[B1] Alzheimer A. (1906). Über einen eigenartigen schweren Erkrankungsprozeβ der Hirnrincle. *Neurol. Cent.* 25:1134.

[B2] Bakovic J. Yu B. Y. K. Silva D. Chew S. P. Kim S. Ahn S. H. (2019). A key metabolic integrator, coenzyme A, modulates the activity of peroxiredoxin 5 via covalent modification. *Mol. Cell. Biochem.* 461 91–102. 10.1007/s11010-019-03593-w 31375973 PMC6790197

[B3] Battisti A. Ciasca G. Grottesi A. Bianconi A. Tenenbaum A. (2012). Temporary secondary structures in tau, an intrinsically disordered protein. *Mol. Simul.* 38 525–533. 10.1080/08927022.2011.633347

[B4] Binder L. I. Frankfurter A. Rebhun L. I. (1985). The distribution of tau in the mammalian central nervous system. *J. Cell Biol*. 101 1371–1378. 10.1083/jcb.101.4.1371 3930508 PMC2113928

[B5] Bourré G. Cantrelle F. X. Kamah A. Chambraud B. Landrieu I. Smet-Nocca C. (2018). Direct crosstalk between O-GlcNAcylation and phosphorylation of tau protein investigated by NMR spectroscopy. *Front. Endocrinol*. 9:595. 10.3389/fendo.2018.00595 30386294 PMC6198643

[B6] Brass E. P. Tahiliani A. G. Allen R. H. Stabler S. P. (1990). Coenzyme A metabolism in vitamin B-12 deficient rats. *J. Nutr.* 120 290–297. 10.1093/jn/120.3.290 2319347

[B7] Burns A. Iliffe S. (2009). Alzheimer’s disease. *BMJ* 338 467–471. 10.1136/bmj.b158 19196745

[B8] Castellano J. M. Deane R. Gottesdiener A. J. Verghese P. B. Stewart F. R. West T. (2012). Low-density lipoprotein receptor overexpression enhances the rate of brain-to-blood Aβ clearance in a mouse model of β-amyloidosis. *Proc. Natl. Acad. Sci. U. S. A*. 109 15502–15507. 10.1073/pnas.1206446109 22927427 PMC3458349

[B9] Cenini G. Voos W. (2019). Mitochondria as potential targets in Alzheimer disease therapy: an update. *Front. Pharmacol*. 10:902. 10.3389/fphar.2019.00902 31507410 PMC6716473

[B10] Chew H. Solomon V. A. Fonteh A. N. (2020). Involvement of Lipids in Alzheimer’s Disease Pathology and Potential Therapies. *Front. Physiol.* 11:598. 10.3389/fphys.2020.00598 32581851 PMC7296164

[B11] Chidambaram H. Chinnathambi S. (2020). Role of cysteines in accelerating tau filament formation. *J. Biomol. Struct. Dyn.* 15 1–10. 10.1080/07391102.2020.1856720 33317395

[B12] Cohen T. J. Friedmann D. Hwang A. W. Marmorstein R. Lee V. M. (2013). The microtubule-associated tau protein has intrinsic acetyltransferase activity. *Nat. Struct. Mol. Biol.* 20 756–762. 10.1038/nsmb.2555 23624859 PMC3827724

[B13] Cox J. Mann M. (2008). MaxQuant enables high peptide identification rates, individualized p.p.b.-range mass accuracies and proteome-wide protein quantification. *Nat. Biotechnol.* 26 1367–1372. 10.1038/nbt.1511 19029910

[B14] Davaapil H. Tsuchiya Y. Gout I. (2014). Signalling functions of coenzyme A and its derivatives in mammalian cells. *Biochem. Soc. Trans*. 42 1056–1062. 25110002 10.1042/BST20140146

[B15] Dusi S. Valletta L. Haack T. B. Tsuchiya Y. Venco P. Pasqualato S. (2014). Exome sequence reveals mutations in CoA synthase as a cause of neurodegeneration with brain iron accumulation. *Am. J. Hum. Genet.* 94 11–22. 10.1016/j.ajhg.2013.11.008 24360804 PMC3882905

[B16] Ferrari L. Rüdiger S. G. D. (2018). Recombinant production and purification of the human protein tau. *Protein Eng. Des. Sel.* 31 447–455. 10.1093/protein/gzz010 31265107

[B17] Fitzpatrick A. W. P. Falcon B. He S. Murzin A. G. Murshudov G. Garringer H. J. (2017). Cryo-EM structures of tau filaments from Alzheimer’s disease. *Nature* 547 185–190. 28678775 10.1038/nature23002 PMC5552202

[B18] Goedert M. (2015). Neurodegeneration. Alzheimer’s and Parkinson’s diseases: the prion concept in relation to assembled Aβ, tau, and α-synuclein. *Science* 349:1255555. 10.1126/science.1255555 26250687

[B19] Goedert M. Spillantini M. G. Cairns N. J. Crowther R. A. (1992). Tau proteins of Alzheimer paired helical filaments: abnormal phosphorylation of all six brain isoforms. *Neuron* 1 159–168. 10.1016/0896-6273(92)90117-v 1530909

[B20] Goedert M. Spillantini M. G. Jakes R. Rutherford D. Crowther R. A. (1989). Multiple isoforms of human microtubule-associated protein tau: sequences and localization in neurofibrillary tangles of Alzheimer’s disease. *Neuron* 3 519–526. 10.1016/0896-6273(89)90210-9 2484340

[B21] Gong C. X. Liu F. Grundke-Iqbal I. Iqbal K. (2005). Post-translational modifications of tau protein in Alzheimer’s disease. *J. Neural Transm*. 112 813–838. 15517432 10.1007/s00702-004-0221-0

[B22] Gout I. (2018). Coenzyme A, protein CoAlation and redox regulation in mammalian cells. *Biochem. Soc. Trans.* 46 721–728. 10.1042/bst20170506 29802218 PMC6008590

[B23] Gout I. (2019). Coenzyme A: a protective thiol in bacterial antioxidant defence. *Biochem. Soc. Trans.* 47 469–476. 10.1042/bst20180415 30783014

[B24] Grundke-Iqbal I. Iqbal K. Tung Y. C. Quinlan M. Wisniewski H. M. Binder L. I. (1986). Abnormal phosphorylation of the microtubule-associated protein tau (tau) in Alzheimer cytoskeletal pathology. *Proc. Natl. Acad. Sci. U. S. A.* 83 4913–4917. 10.1073/pnas.83.13.4913 3088567 PMC323854

[B25] Guo C. Sun L. Chen X. Zhang D. (2013). Oxidative stress, mitochondrial damage and neurodegenerative diseases. *Neural Regen. Res.* 8 2003–2014. 25206509 10.3969/j.issn.1673-5374.2013.21.009 PMC4145906

[B26] Hanger D. P. Byers H. L. Wray S. Leung K. Y. Saxton M. J. Seereeram A. (2007). Novel phosphorylation sites in tau from Alzheimer brain support a role for casein kinase 1 in disease pathogenesis. *J. Biol. Chem.* 282 23645–23654. 10.1074/jbc.m703269200 17562708

[B27] Hashimoto M. Rockenstein E. Crews L. Masliah E. (2003). Role of protein aggregation in mitochondrial dysfunction and neurodegeneration in Alzheimer’s and Parkinson’s diseases. *Neuromolecular. Med*. 4 21–36. 10.1385/nmm:4:1-2:21 14528050

[B28] Hoover B. R. Reed M. N. Su J. Penrod R. D. Kotilinek L. A. Grant M. K. (2010). tau mislocalization to dendritic spines mediates synaptic dysfunction independently of neurodegeneration. *Neuron* 68 1067–1081. 10.1016/j.neuron.2010.11.030 21172610 PMC3026458

[B29] Huang W. J. Zhang X. Chen W. W. (2016). Role of oxidative stress in Alzheimer’s disease. *Biomed. Rep*. 4 519–522. 27123241 10.3892/br.2016.630 PMC4840676

[B30] Iqbal K. Liu F. Gong C. X. (2016). Tau and neurodegenerative disease: the story so far. *Nat. Rev. Neurol*. 12 15–27. 10.1038/nrneurol.2015.225 26635213

[B31] Iqbal K. Liu F. Gong C.-X. Grundke-Iqbal I. (2010). tau in Alzheimer Disease and Related tauopathies. *Curr. Alzheimer Res.* 7 656–664. 10.2174/156720510793611592 20678074 PMC3090074

[B32] Kadavath H. Jaremko M. Jaremko L. Biernat J. Mandelkow E. Zweckstetter M. (2015). Folding of the tau protein on microtubules. *Angew. Chem. Int. Ed. Engl.* 54 10347–10351. 10.1002/anie.201501714 26094605

[B33] Kinney J. W. Bemiller S. M. Murtishaw A. S. Leisgang A. M. Salazar A. M. Lamb B. T. (2018). Inflammation as a central mechanism in Alzheimer’s disease. *Alzheimers Dement.* 4 575–590. 10.1016/j.trci.2018.06.014 PMC6214864 30406177

[B34] Kovacs G. G. (2015). Invited review: neuropathology of tauopathies: principles and practice. *Neuropathol. Appl. Neurobiol*. 41 3–23. 25495175 10.1111/nan.12208

[B35] Landino L. M. Robinson S. H. Skreslet T. E. Cabral D. M. (2004). Redox modulation of tau and microtubule-associated protein-2 by the glutathione/glutaredoxin reductase system. *Biochem. Biophys. Res. Commun.* 323 112–117. 10.1016/j.bbrc.2004.08.065 15351709

[B36] Lee K. H. Cha M. Lee B. H. (2020). Neuroprotective effect of antioxidants in the brain. *Int. J. Mol. Sci.* 21:7152. 10.3390/ijms21197152 32998277 PMC7582347

[B37] Lee V. M. Goedert M. Trojanowski J. Q. (2001). Neurodegenerative tauopathies. *Annu. Rev. Neurosci*. 24 1121–1159. 11520930 10.1146/annurev.neuro.24.1.1121

[B38] Leonardi R. Zhang Y. M. Rock C. O. Jackowski S. (2005). Coenzyme A: back in action. *Prog. Lipid Res.* 44 125–153. 15893380 10.1016/j.plipres.2005.04.001

[B39] Lindwall G. Cole R. D. (1984). Phosphorylation affects the ability of tau protein to promote microtubule assembly. *J. Biol. Chem.* 259 5301–5305. 10.1016/s0021-9258(17)42989-9 6425287

[B40] Lipmann F. Kaplan N. O. (1946). A common factor in the enzymatic acetylation of sulfanilamide and of choline. *J. Biol. Chem*. 162 743–744. 10.1016/s0021-9258(17)41419-0

[B41] Liu F. Gong C. X. (2008). tau exon 10 alternative splicing and tauopathies. *Mol. Neurodegener*. 3:8. 10.1186/1750-1326-3-8 18616804 PMC2483273

[B42] Liu F. Iqbal K. Grundke-Iqbal I. Hart G. W. Gong C.-X. (2004). O-GlcNAcylation regulates phosphorylation of tau: a mechanism involved in Alzheimer’s disease. *Proc. Natl. Acad. Sci. U. S. A*. 101 10804–10809. 10.1073/pnas.0400348101 15249677 PMC490015

[B43] Liu F. Shi J. Tanimukai H. Gu J. Grundke-Iqbal I. Iqbal K. (2009). Reduced O-GlcNAcylation links lower brain glucose metabolism and tau pathology in Alzheimer’s disease. *Brain J. Neurol.* 132 1820–1832. 10.1093/brain/awp099 19451179 PMC2702834

[B44] Magi S. Castaldo P. Macrì M. Maiolino M. Matteucci A. Bastioli G. (2016). Intracellular Calcium Dysregulation: implications for Alzheimer’s Disease. *Biomed. Res. Int.* 2016 1–14. 10.1155/2016/6701324 PMC4909906 27340665

[B45] Malanchuk O. M. Panasyuk G. G. Serbyn N. M. Gout I. T. Filonenko V. V. (2015). Generation and characterization of monoclonal antibodies specific to Coenzyme A. *Biopolym. Cell* 31 187–192. 10.7124/bc.0008df

[B46] Mandal A. Drerup C. M. (2019). Axonal transport and mitochondrial function in neurons. *Front. Cell. Neurosci*. 13:373. 10.3389/fncel.2019.00373 31447650 PMC6696875

[B47] Martin L. Latypova X. Terro F. (2011). Post-translational modifications of tau protein: implications for Alzheimer’s disease. *Neurochem. Int.* 58 458–471. 10.1016/j.neuint.2010.12.023 21215781

[B48] Martinho M. Allegro D. Huvent I. Chabaud C. Eteinnel E. Kovacic H. (2018). Two tau binding sites on tubulin revealed by thiol-disulfde exchanges. *Sci. Rep*. 8:13846. 10.1038/s41598-018-32096-9 PMC6138654 30218010

[B49] McAllister R. A. Fixter L. M. Campbell E. H. G. (1988). The effect of tumour growth on liver pantothenate, CoA, and fatty acid synthetase activity in the mouse. *Br. J. Cancer* 57 83–86. 10.1038/bjc.1988.14 2831928 PMC2246678

[B50] Meraz-Rios M. A. Lira-De Leon L. Campos-Pena V. De Anda-Hernandez M. Mena-Lopez R. (2010). Tau oligomers and aggregation in alzheimer’s disease. *J. Neurochem.* 112 1353–1367. 19943854 10.1111/j.1471-4159.2009.06511.x

[B51] Miao J. Shi R. Li L. Chen F. Zhou Y. Tung Y. C. (2019). Pathological tau from alzheimer’s brain induces site-specific hyperphosphorylation and SDS- and reducing agent-resistant aggregation of tau in vivo. *Front. Aging Neurosci.* 11:34. 10.3389/fnagi.2019.00034 30890929 PMC6411797

[B52] Montine T. J. Phelps C. H. Beach T. G. Bigio E. H. Cairns N. J. Dickson D. W. (2012). National Institute on Aging-Alzheimer’s Association guidelines for the neuropathologic assessment of Alzheimer’s disease: a practical approach. *Acta Neuropathol*. 123 1–11. 22101365 10.1007/s00401-011-0910-3 PMC3268003

[B53] Morishima-Kawashima M. Hasegawa M. Takio K. Suzuki M. Yoshida H. Titani K. (1995). Proline-directed and non-proline-directed phosphorylation of PHF-tau. *J. Biol. Chem*. 270 823–829. 10.1074/jbc.270.2.823 7822317

[B54] Nixon R. A. Yang D. S. (2012). Autophagy and neuronal cell death in neurological disorders. *Cold Spring Harb. Perspect. Biol*. 4:a008839. 10.1101/cshperspect.a008839 22983160 PMC3475163

[B55] Przedborski S. Vila M. Jackson-Lewis V. (2003). Neurodegeneration: what is it and where are we? *J. Clin. Invest*. 111 3–10. 12511579 10.1172/JCI17522 PMC151843

[B56] Ramkumar A. Jong B. Y. Ori-McKenney K. M. (2018). ReMAPping the microtubule landscape: how phosphorylation dictates the activities of microtubule-associated proteins. *Dev. Dyn.* 247 138–155. 10.1002/dvdy.24599 28980356 PMC5739964

[B57] Reibel D. K. Wyse B. W. Berkich D. A. Neely J. R. (1981). Regulation of coenzyme A synthesis in heart muscle: effects of diabetes and fasting. *Am. J. Physiol.* 240 H606–H611. 7013504 10.1152/ajpheart.1981.240.4.H606

[B58] Ross C. A. Poirier M. A. (2004). Protein aggregation and neurodegenerative disease. *Nat. Med.* 10 S10–S17. 15272267 10.1038/nm1066

[B59] Schweers O. Mandelkow E. M. Biernat J. Mandelkow E. (1995). Oxidation of cysteine-322 in the repeat domain of microtubule-associated protein tau controls the *in vitro* assembly of paired helical filaments. *Proc. Natl. Acad. Sci. U. S. A.* 92 8463–8467. 10.1073/pnas.92.18.8463 7667312 PMC41177

[B60] Selkoe D. J. Lansbury P. J. Jr. (1999). *Alzheimer’s Disease is The Most Common Neurodegenerative Disorder (Basic Neurochemistry: Molecular, Cellular and Medical Aspects).* Philadelphia, PA: Lippincott-Raven.

[B61] Soto C. (2003). Unfolding the role of protein misfolding in neurodegenerative diseases. *Nat. Rev. Neurosci.* 4 49–60. 10.1038/nrn1007 12511861

[B62] Srinivasan B. Sibon O. C. M. (2014). Coenzyme A, more than ‘just’ a metabolic cofactor. *Biochem. Soc. Trans.* 42 1075–1079. 10.1042/bst20140125 25110005

[B63] Stoker M. L. Newport E. Hulit J. C. West A. P. Morten K. J. (2019). Impact of pharmacological agents on mitochondrial function: a growing opportunity? *Biochem. Soc. Trans.* 47 1757–1772. 10.1042/bst20190280 31696924 PMC6925523

[B64] Sultana R. Perluigi M. Butterfield D. A. (2009). Oxidatively modified proteins in Alzheimer’s disease (AD), mild cognitive impairment and animal models of AD: role of Abeta in pathogenesis. *Acta Neuropathol.* 118 131–150. 10.1007/s00401-009-0517-0 19288120 PMC2818870

[B65] Theodoulou F. L. Sibon O. C. M. Jackowski S. Gout I. (2014). Coenzyme A and its derivatives: renaissance of a textbook classic. *Biochem. Soc. Trans*. 42 1025–1032. 10.1042/bst20140176 25109997

[B66] Tossounian M. A. Zhang B. Gout I. (2020). The writers, readers, and erasers in redox regulation of GAPDH. *Antioxidants* 9:1288. 10.3390/antiox9121288 33339386 PMC7765867

[B67] Tsuchiya Y. Byrne D. P. Burgess S. G. Bormann J. Bakovic J. Huang Y. (2020). Covalent Aurora A regulation by the metabolic integrator coenzyme A. *Redox Biol.* 28:101318. 10.1016/j.redox.2019.101318 31546169 PMC6812009

[B68] Tsuchiya Y. Peak-Chew S. Y. Newell C. Miller-Aidoo S. Mangal S. Zhyvoloup A. (2017). Protein CoAlation: a redox-regulated protein modification by coenzyme A in mammalian cells. *J. Biochem.* 474 2489–2508. 10.1042/bcj20170129 28341808 PMC5509381

[B69] Tsuchiya Y. Zhyvoloup A. Bakovicì J. Thomas N. Yu B. Das S. (2018). Protein CoAlation and antioxidant function of coenzyme A in prokaryotic cells. *J. Biochem.* 475 1909–1937. 10.1042/bcj20180043 29626155 PMC5989533

[B70] Vasili E. Dominguez-Meijide A. Fleming-Outeiro T. (2019). Spreading of α-Synuclein and tau: a systematic comparison of the mechanisms involved. *Front. Mol. Neurosci.* 12:107. 10.3389/fnmol.2019.00107 31105524 PMC6494944

[B71] Von Bergen M. Friedhoff P. Biernat J. Heberle J. Mandelkow E.-M. Mandelkow E. (2000). Assembly of tau protein into Alzheimer paired helical filaments depends on a local sequence motif ((306)VQIVYK(311)) forming beta structure. *Proc. Natl. Acad. Sci. U. S. A.* 97 5129–5134. 10.1073/pnas.97.10.5129 10805776 PMC25793

[B72] Wang J. Z. Xia Y. Y. Grundke-Iqbal I. Iqbal K. (2013). Abnormal hyperphosphorylation of tau: sites, regulation, and molecular mechanism of neurofibrillary degeneration. *J. Alzheimers Dis.* 33 S123–S139. 22710920 10.3233/JAD-2012-129031

[B73] Wang W. Zhao F. Ma X. Perry G. Zhu X. (2020). Mitochondria dysfunction in the pathogenesis of Alzheimer’s disease: recent advances. *Mol. Neurodegener.* 15:30. 10.1186/s13024-020-00376-6 PMC7257174 32471464

[B74] Wesseling H. Mair W. Kumar M. Schlaffner C. N. Tang S. Beerepoot P. (2020). tau PTM profiles identify patient heterogeneity and stages of Alzheimer’s disease. *Cell* 183 1699–1713. 10.1016/j.cell.2020.10.029 33188775 PMC8168922

[B75] Yang X. J. Seto E. (2008). Lysine acetylation: codified crosstalk with other posttranslational modifications. *Mol. Cell* 31 449–461. 10.1016/j.molcel.2008.07.002 18722172 PMC2551738

[B76] Yiannopoulou K. G. Papageorgiou S. G. (2020). Current and Future Treatments in Alzheimer Disease: an Update. *J. Cent. Nerv. Syst. Dis.* 12:1179573520907397. 10.1177/1179573520907397 PMC7050025 32165850

[B77] Yu B. Y. K. Tossounian M. A. Hristov S. D. Lawrence R. Arora P. Tsuchiya Y. (2021). Regulation of metastasis suppressor NME1 by a key metabolic cofactor coenzyme A. *Redox Biol.* 44:101978. 10.1016/j.redox.2021.101978 33903070 PMC8212152

[B78] Zhou B. Westaway S. K. Levinson B. Johnson M. A. Gitschier J. Hayflick S. J. (2001). A novel pantothenate kinase gene (PANK2) is defective in Hallervorden-Spatz syndrome. *Nat. Genet*. 28 345–349. 10.1038/ng572 11479594

