# Supplementary material for: Extensive Anti-CoA Immunostaining in Alzheimer’s Disease and Covalent Modification of Tau by a Key Cellular Metabolite Coenzyme A
Source: Front Cell Neurosci. 2021 Oct 15;15:739425. doi: 10.3389/fncel.2021.739425 (PMC8554225; doi:10.3389/fncel.2021.739425)
Supplement: Supplementary file 1 [file Data_Sheet_1.PDF]

Supplementary Table 1. Extended case demographic data for the post-mortem cases used in the study.

Case ID	AAO (years)	AAD (years)	DD (years)	Sex	Path Diagnosis	PM delay (hours)	Brain Weight (g)	Braak Tau	Thal	CERAD	ABC score
1	59	74	15	F	AD	102:55:00	1265	6	4	3	A3B3C3
2	59	76	17	F	AD	60:25:00	1191	6	5	3	A3B3C3
3	60	71	11	M	AD	52:39:00	1327	6	3	3	A2B3C3
4	74	79	5	F	AD	60:40:00	1200	6	5	3	A3B3C3
5	52	68	16	M	AD	73:45:00	1234	6	5	3	A3B3C3
6	55	67	12	M	AD	28:35:00	1015	6	5	3	A3B3C3
7	72	88	16	M	AD	58:10:00	1084	6	5	2	A3B3C2
8	63	73	10	M	AD	31:10:00	1269	6	5	3	A3B3C3
9	49	62	13	F	AD	76:40:00	996	6	5	3	A3B3C3
10	63	74	11	M	AD	33:26:00	1022	6	5	3	A3B3C3
11	48	61	13	M	AD	40:40:00	1650	6	5	3	A3B3C3
12	58	72	14	F	AD	81:26:00	820	6	5	3	A3B3C3
13	48	63	15	M	AD	31:42:00	1042	6	5	3	A3B3C3
14	51	69	18	F	AD	81:00:00	1056	6	5	3	A3B3C3
15	54	65	11	M	AD	34:25:00	1089	6	5	3	A3B3C3
16	na	103	na	F	Control	26:35:00	975	4	5	1	A3B2C1
17	na	88	na	M	Control	27:30:00	1330	4	3	2	A2B2C2
18	na	83	na	M	Control	105:28:00	1244	4	3	2	A2B2C2
19	na	92	na	M	Control	46:15:00	1213	4	3	2	A2B2C2
20	na	91	na	F	Control	69:20:00	1311	4	4	2	A3B2C2
21	60	68	8	F	PSP	36:50:00	1177	1	1	0	A1B1C0
22	75	84	9	F	PSP	70:00:00	1095	0	3	1	A2B0C1
23	76	84	8	M	PSP	50:00:00	1370	0	4	1	A3B0C1
24	57	62	5	M	PSP	72:20:00	1369	5	5	2	A3B3C2
25	66	79	13	F	PSP	73:55:00	1141	0	3	1	A2B0C1
26	58	65	7	M	CBD	48:04:00	1232	0	0	0	A0B0C0
27	54	61	7	M	CBD	102:30:00	1389	0	0	0	A0B0C0
28	58	69	11	F	CBD	103:15:00	917	0	0	0	A0B0C0
29	57	64	7	M	CBD	41:25:00	1456	0	0	0	A0B0C0
30	63	69	6	M	CBD	81:36:00	1291	0	2	1	A1B0C1
31	60	84	24	M	PD	71:05:00	1484	2	1	0	A1B1C0
32	80	89	9	M	PD	26:45:00	1493	4	4	2	A3B2C2
33	69	78	9	M	PD	95:15:00	1600	2	5	2	A3B1C2
34	77	83	6	M	PD	96:25:00	1644	1	3	1	A2B1C1
35	65	78	13	F	PD	84:50:00	1203	1	1	0	A1B1C0
36	67	75	8	M	MSA	54:00:00	1359	1	1	0	A1B1C0
37	46	52	6	F	MSA	79:00:00	1354	1	0	0	A0B1C0
38	33	42	9	M	MSA	30:10:00	1380	0	0	0	A0B0C0
39	63	72	9	M	MSA	82:00:00	1450	1	0	0	A0B1C0
40	57	63	6	M	MSA	102:55:00	1234	0	2	0	A1B0C0
